# Familial liability, obstetric complications and childhood development abnormalities in early onset schizophrenia: a case control study

**DOI:** 10.1186/1471-244X-11-60

**Published:** 2011-04-14

**Authors:** Francesco Margari, Maria G Petruzzelli, Paola A Lecce, Orlando Todarello, Andrea De Giacomo, Elisabetta Lucarelli, Domenico Martinelli, Lucia Margari

**Affiliations:** 1 Department of Neurologic and Psychiatric Sciences, Psychiatric Unit, University of Bari, Bari, Italy; 2 Department of Neurologic and Psychiatric Sciences, Child Neuropsychiatric Unit, University of Bari, Bari, Italy; 3 Department of medical and occupational science (DIMED), section of Hygiene, University of Foggia, Foggia, Italy

## Abstract

**Background:**

Genetic and environmental risk factors and gene-environment interactions are linked to higher likelihood of developing schizophrenia in accordance with the neurodevelopmental model of disease; little is known about risk factors and early development in early-onset schizophrenia (EOS) and very early-onset schizophrenia (VEOS).

**Methods:**

We present a case-control study of a sample of 21 patients with EOS/VEOS and a control group of 21 patients with migraine, recruited from the Child Neuropsychiatry Unit, Department of Neurologic and Psychiatric Science, University of Bari, Italy. The aim was to assess the statistical association between VEOS/EOS and family history for psychiatric disorders, obstetric complications and childhood developmental abnormalities using 2 × 2 tables and a Chi Squared or Fisher test.

**Results:**

The results show a statistical association between EOS/VEOS and schizophrenia and related disorders (P = 0.02) and personality disorders (P = 0.003) in relatives, and between EOS/VEOS and developmental abnormalities of early relational skills (P = 0.008) and learning (P = 0.04); there is not a statistically relevant difference between cases and controls (P > 0.05) for any obstetric complications (pre, peri and postpartum).

**Conclusions:**

This study confirms the significant role of familial liability but not of obstetric complications in the pathogenesis of VEOS/EOS; the association between childhood developmental abnormalities and EOS/VEOS supports the neurodevelopmental model of disease.

## Background

Early-onset schizophrenia (EOS), manifesting before the age of 18 years, and very early-onset schizophrenia (VEOS), developing before the age of 13 years, are considered more severe and uncommon variants of the adult-onset disorder; these clinical forms may be related to a greater vulnerability by reason of higher risk factors for schizophrenia and may be preceded by more relevant neurodevelopmental abnormalities than the adult onset form of the illness [1-4].

The study of childhood-onset schizophrenia conducted by the National Institute of Mental Health (NIMH) revealed more severe premorbid neurodevelopmental abnormalities, a higher rate of cytogenetic anomalies, and a seemingly higher rate of familial schizophrenia spectrum disorders than in later onset cases; there was no evidence of increased obstetric complications or environmental stress [5,6]. Vourdas et al. found that developmental deviance and premorbid abnormalities of social interaction and language-related functions tend to lead to a more precocious onset of schizophrenia [7].

On the other hand, information obtained by parents about risk factors for schizophrenia and early neurodevelopment should be more recent and more reliable for children and adolescents patients than adult onset patients; therefore retrospective studies could lead to more consistent data in case of EOS and VEOS rather than in case of adult onset schizophrenia.

In the present study we examined a sample of 21 patients with EOS and VEOS; the control group was composed of 21 non-psychiatric patients affected by migraine, supposed to have a different pathogenesis for the illness; comparison was made according to age and gender. The aim of the study was the following:

1. To carry out a retrospective analysis of the frequency and typology of familial liability for psychiatric disorder, obstetric complications and childhood developmental abnormalities in the cases and controls;

2. To verify statistical association between EOS/VEOS and familial and environmental risk factors and childhood developmental abnormalities with respect to the control group.

## Methods

### a) Subjects

The study sample consisted of 21 patients of both sexes, with a diagnosis of schizophrenia in accordance with the general diagnostic criteria of the Diagnostic and Statistical Manual for Mental Disorders, IV Edition-Text Revision DSM IV-TR [8], with the onset of psychotic symptoms before the age of 18 years (EOS) or before the age of 13 years (VEOS). They were recruited over a three-year period from the Child Neuropsychiatry Unit, Department of Neurologic and Psychiatric Science, University of Bari, Italy.

The control sample consisted of 21 patients of both sexes, with a diagnosis of migraine in accordance with the International Classification of Headache Disorders-II edition, ICHD-II [9], followed clinically over the last year from the Child Neuropsychiatry Unit, Department of Neurologic and Psychiatric Science, University of Bari too. They were matched to the EOS/VEOS patients according to age and gender; exclusion criteria were evidence of a psychiatric comorbidity.

The study was approved by the local ethical committee; all the parents who were interviewed provided written consent.

### b) Assessment

The diagnosis of schizophrenia and migraine was made by an experienced child neuropsychiatrist on the basis of interviews with the child and the family, a review of past clinical records and historical information, and was supported by the Kiddie-Schedule for Affective Disorders and Schizophrenia-Present and Lifetime Version, K-SADS-PL [10] and Child Behaviour Checklist, CBCL [11].

A comprehensive diagnostic assessment was made in both cases and controls, including a physical, neurological and psychological examination and an instrumental evaluation by electroencephalograph (EEG) and brain magnetic resonance images (MRI). All patients were studied with Wechsler Intelligence Scale for Children-Revised, WISC-R [12] for the evaluation of Intelligence Quotient (IQ).

Data about a family history of psychiatric diseases were collected using the family history interview method [13]. At least one family member, usually one of the parents, was interviewed to inquire about both first and second-degree relatives; whenever possible at least one other family member was also contacted to maximize the accuracy of the information. A positive or negative family history was used as a dichotomous indicator of familial loading for schizophrenia and related disorders, affective disorders, anxiety disorders, substances abuse/dependence, personality disorders, and any unspecified mental disorder.

After examining the relevant documents showing precocious environmental risk factors for schizophrenia [14-22], a list was drawn up of the main obstetric complications that may be involved in the development of the disease. Three groups of complications were investigated: prepartum complications (bleeding, diabetes, rhesus incompatibility, preeclampsia, anemia, toxemia, placental abruption, smoking, drinking or drug addiction, any medicament taken, maternal infections, psychological maternal stress, threatened premature delivery), peripartum complications (birth weight < 2000 g, emergency cesarean section, fetal hypoxia or anoxia, gestational age < 37 weeks or > 42 weeks, delivery by forceps or with cord around neck, labour < 3 h or > 36 h), postpartum complications (drugs given during the neonatal period, early infections, continued hospitalization of the baby after the discharge of the mother). Information about these precocious environmental risk factors was collected by interviewing the parents and examining birth records.

Using a modified version of Developmental Scale [7] with addiction to collect other information, the parents were also asked about the presence of any deviations in neuropsychological development during the first few years of life, with particular reference to the development of motor skills, language abilities, autonomous sphincter control, early relational skills, school progress,

### c) Statistical analysis

To assess the correlations between VEOS/EOS and familial liability, obstetric complications and childhood developmental abnormalities 2 × 2 tables were constructed and a Chi Squared or Fisher test was calculated. When it was possible, OR (Odds Ratio) and its 95% CI (Confidential Interval) were reckoned. Chosen significance level was selected as P < 0.05. Statistical analysis was performed by Stata MP for Mac Os 10.

## Results

### a) Demographic and clinical features of cases and controls

The main demographic features of cases and controls are resumed in table 1.

**Table 1:** Demographic features of cases and controls

	Cases N 21	Controls N 21
Mean age	11	11
Range of age	7-16	7-16
Sex		
Male	12	10
Female	9	11

In the study sample there were 5 cases of EOS and 16 cases of VEOS; the mean age of onset of psychotic symptoms was 10 years. There was no significant difference in the mean age of onset according to gender (10 years for males, 11 years for females), although there was a wider range in males, from 5 to 15 years as compared to the female range from 9 to 14 years.

All the EOS/VEOS subjects presented with nonspecific prodromic symptoms. In the great majority of cases (80%) the onset of psychotic symptoms was gradual and insidious. Manifest disturbances were mostly in the form of negative symptoms(71%), followed by delusions (43%) and disorganized behaviour (43%); in 33% of the cases there were hallucinations, equally frequent visual and auditory; disorganized speech was present in the same percentage of 33%.

Further to DSM criteria, assessment of the intelligence quotient showed that mild mental delay was present in 24% of the cases, while 9.5% had a borderline IQ.

EEG abnormalities were found in 38% of the cases, the most common findings being focal slow activity and focal paroxysmal activity (i.e. spikes, sharp waves or complexes). However, no specific EEG pattern emerged. A single case was affected by the clinical condition of "epileptic encephalopathy with polymorphic crises".

Nonspecific alterations to MRI were found in 24% of the cases, consisting of mild gliotic damage probably attributable to pre and perinatal parenchymal hypoxia. In one case proton magnetic resonance spectroscopy (1H-MRS) showed an elevated lipids peak in both frontal regions with normal values of N-acetylaspartate (NAA), creatine plus phosphocreatine (Cr) and choline (Cho) and NAA/Cr and Cho/Cr ratios; the full clinical and neuroimaging study of this patient is described in Margari F. et al, 2008 [2]. In another patient 1H-MRS showed a mild alteration to the Cho/Cr ratio due to an increased choline peak of the white matter of the semioval centers.

One of the females with a diagnosis of VEOS was adopted, so it was not possible to collect data on familial psychiatric disturbances and neither early environmental damage.

All the controls showed a normal IQ; in none of them EEG/MRI alterations were shown to occur.

### b) Familial liability

The data collected on family history for psychiatric disorders for both groups of cases and controls are presented in table 2. As we expected, there was a significant statistical association between psychiatric disease in relatives and risk for developing schizophrenia (OR: 6.5, 95% CI: 1.3-35.1; P = 0.0074). Specifically, there was a statistical association between EOS/VEOS and schizophrenia and related disorders (P = 0.02) and personality disorders (P = 0.003) in relatives. Moreover we noted that in 50% of the cases of EOS/VEOS familial liability was present in both parents' families; anxiety and personality disorders were shown to affect only the probands' parents; mood disturbances, drug abuse and unspecified psychiatric disturbances concerned both the parents and other relatives; schizophrenia and other psychotic disturbances were shown to affect second degree relatives only.

**Table 2:** Family history of psychiatric disorders in EOS/VEOS and control relatives

	Cases N (%)	Controls N (%)	Total N (%)	OR (95% CI)	P value
Family history of psichiatric disorders	16 (80%)	8 (38%)	24 (41%)	6.5(1.3-35.1)	0.0074
Schizophrenia and related disorders	5 (25%)	0	5 (12%)	___	0.0207
Affective disorders	5 (25%)	2 (9%)	7 (17%)	3.2(0.4-36.6)	0.1880
Anxiety disorders	6 (30%)	5 (24%)	11 (27%)	1.4(0.3-7.1)	0.6547
Personality disorders	7 (35%)	0	7 (35%)	___	0.0029
Substance abuse/dependence	2 (10%)	0	2 (10%)	___	0.2317
Unspcified mental disorders	3 (15%)	1 (4%)	4 (9%)	3.5(0.2-194.3)	0.2694

### c) Obstetric complications

Exposure to early environmental risk factors had occurred in 55% of the subjects with VEOS/EOS; the detailed description of the obstetric complications reported in the cases of VEOS/EOS with a history of early exposure to environmental risks are resumed in table 3. The 43% of the control patients with migraine had a history of obstetric complications. The difference between cases and controls was not statistically relevant (P > 0.05) for any obstetric complications (prepartum, peripartum and postpartum complications), as reported in table 4.

**Table 3:** Pre-, peri- and postpartum complications in EOS/VEOS subjects with history of obstetric complications

Prepartum		Peripartum		Postpartum	
Bleeding	10%	Emergency cesarean section	50%	Drugs	75%
Placental abruption	20%	Fetal hipoxia	67%	Early infections	75%
Psychological maternal stress	30%	Gestational age > 37 weeks	33%	Continued hospitalization	25%
Threatened premature delivery	70%	Delivery by forceps	17%	-----	---

**Table 4:** Obstetric complications in EOS/VEOS and controls

	Cases N (%)	Controls N (%)	Total N (%)	OR (95% CI)	P value
Obstetric complications	11 (55%)	9 (43%)	20 (48%)	1.6(0.4-6.7)	0.4369
					
Prepartum	10 (50%)	7 (33%)	17 (41%)	2(0.5-8.5)	0.2789
Peripartum	6 (30%)	4 (20%)	10 (24%)	1.8(0.3-10.5)	0.4143
Postpartum	4 (20%)	0	4 (20%)	___	0.0310

### d) Childhood developmental abnormalities

Childhood developmental abnormalities were present in 65% of the subjects with EOS/VEOS: the most commonly involved areas were school progress, reported in 46% of the cases and early relational skills, reported in 46% of the cases. In 31% of the cases disturbances in the development of language abilities were observed, and enuresis was present in the same percentage. No problems of motor skills were recorded. There was a significant statistical association between childhood developmental abnormalities and EOS/VEOS (OR: 37.1, 95% CI: 3.8-1654.1; P < 0.001). Specifically, there was a statistical association between EOS/VEOS and developmental abnormalities of relational skills (P = 0.008) and learning (P = 0.04). The data about childhood developmental abnormalities are presented in table 5.

**Table 5:** Childhood developmental abnormalities in EOS/VEOS and controls

	Cases N (%)	Controls N (%)	Total N (%)	OR (95% CI)	P value
Childhood developmental abnormalities	13 (65%)	1 (4%)	14 (34%)	37.1(3.8-1654.0)	0.0000
motor skills	0	0	0	___	___
language abilities	5 (25%)	1 (4%)	6 (14%)	6.6(0.6-330.6)	0.0931
sphincter control	4 (20%)	0	4 (9%)	___	0.0310
relational skills	6 (30%)	0	6 (14%)	___	0.0066
school progress	6 (30%)	0	6 (14%)	___	0.0086

## Discussion

In accordance with neurodevelopmental studies, schizophrenia can be seen as a disorder with age-dependent clinical manifestations that in a minority of individuals start during childhood [23-28]. Although the prevalence of EOS has not been adequately studied, the American Academy of Child and Adolescent Psychiatry suggest that EOS, and especially VEOS, are predominantly observed in males, with a ratio of approximately 2:1 and that with increasing age, this ratio tends to even out [1]. In our sample there were no significant sex differences as regards either prevalence or mean age of onset. Konnecke et al. have claimed that the difference in the age of onset between men and women is considerably reduced in the presence of a strong genetic vulnerability to schizophrenia and a history of pre and perinatal complications [29]. In 80% of our sample of children with EOS and VEOS we found a familial history of psychiatric disorders with a statistically relevant difference between cases and controls. This confirms the important role of familial loading as a risk factor for schizophrenia, being comparable to the data on adult onset forms [30-32]. The first family study of childhood-onset schizophrenia was published by Asarnow et al; they found that relatives of probands with childhood-onset disease had an increased lifetime morbid risk for schizophrenia and schizotypal personality disorder as compared to the relatives of children and adolescents with attention deficit hyperactivity disorder and to the relatives of community comparison subjects [33]. Nicolson et al. found a greater morbid risk for schizophrenia spectrum disorders (schizophrenia, schizoaffective disorder, other nonaffective psychotic disorders, schizotypal personality disorder, paranoid personality disorder) in the parents of patients with childhood onset than with adult onset schizophrenia and both these groups were at a higher risk than the parents of community comparison subjects. Nevertheless, in their study, schizophrenia was an uncommon diagnosis in all three groups of parents; no diagnosis of either a schizoaffective disorder or another nonaffective psychotic disorder was present in any of the groups; moreover the parents of patients with childhood onset schizophrenia had a greater morbid risk for a schizotypal personality disorder than the other two groups and for a paranoid personality disorder than the parents of community comparison subjects [34]. In this study we found that the relatives of VEOS/EOS patients differed significantly from the relatives of control subjects in terms of family history of schizophrenia and related disorders and of personality disorders. As reported by Nicolson et al., in this sample too, the parents of VEOS/EOS patients had only diagnosis of personality disorders and never of schizophrenia and related disorders. As reported in other studies on familial liability for schizophrenia, we found that EOS/VEOS is associated with a specific increase in family history for schizophrenia and related disorders as well as for personality disorders, rather than general psychopathology. Furthermore, the different psychopathological expression between first and second degree relatives suggests that we should widen our knowledge about the personality of EOS/VEOS parents. As matter of fact psychopathological traits such as suspiciousness, withdrawal, social avoidance, introversion, diffidence, flattened affectivity are likely to account for the phenotypical expression relevant to familial vulnerability to schizophrenia, characterized by both genetic and environmental factors [35,36].

While familial risk factors account for a significant rate of predisposition to schizophrenia, there is evidence of an important environmental contribution [17,32]. Obstetric complications are among the most studied environmental indicators of risk for schizophrenia although discordant data have also been reported about the effective pathogenetic role in schizophrenia disease also because current methods of investigating the relationship between obstetric complication and schizophrenia are reaching the limit of their usefulness [37,18,19,38,22]. The consequences of obstetric complications lack diagnostic specificity according to the level of hypoxemic stress suffered and to the genetic predisposition of the foetus [14,39]. There is poor consensus about the pathogenetic mechanisms through which pre and perinatal damage can favour the development of schizophrenia. Rosso et al. proposed a model whereby the neurotoxic effects of fetal hypoxia can trigger early onset of schizophrenia due to premature cortical synaptic pruning [21]. Our data show the presence of environmental risk factors in 55% of the sample, a higher proportion than the literature data on the frequency of obstetric complications in patients with adult onset schizophrenia (7-20% according to Boog G [14], 21.5-31.7% according to Nicolson et al. [6]). Other Authors have also supported this association between obstetric complication and an increased risk for early onset schizophrenia [15,20-22]. Moreover, most of the obstetric complications reported in our sample were correlated to fetal hypoxia (bleeding, placental abruption, threatened premature delivery, peripartum fetal hypoxia, emergency cesarean section, forceps delivery). This notwithstanding, as Ordonez et al. in 2005 [40], we didn't find either a relevant association between obstetric complications and VEOS/EOS when compared the patients to control subjects; then we can't support the role of obstetric complications as risk factors of schizophrenia, also when examined more specifically as pre-, peri- and postpartum complications. On the other hand the nature and strength of the association between obstetric complications and schizophrenia has been questioned yet [16] and the hypothesis that exposure to obstetric complications may interact with a genetic liability and increased the vulnerability to schizophrenia remains difficult to assess [39].

Studies of adult onset schizophrenia have demonstrated that developmental delay, early functional impairment and aspecific psychopathologic symptoms are often observed prior to the full emergence of psychotic symptoms [24,41,26,27,43]. They may be considered as a part of a longitudinal psychotic phenotype in which some aspects are already established in early life; alternatively, the effects may reveal a greater vulnerability depending on exposure to further causes, but which may also be susceptible to protective factors. It is unclear whether neurodevelopmental abnormalities are on the increase in patients with early onset schizophrenia, nor whether do they act to precipitate the earlier onset of the disorder[24,31,7]. We found a significant statistical association between childhood developmental abnormalities and the risk for schizophrenia, particularly affecting relational skills and learning. Moreover, about one third of our sample showed a low IQ. The prevalence of mental disturbances is known to be approximately four-fold in mentally retarded subjects as compared to the general population, although the data on prevalence by single diagnostic category are less precise [44,45]. It is generally agreed that cognitive deficits play an important role in the malfunctioning mechanism underlying the disorder [46]. Cognitive deficits have been documented in practically every domain, being most pronounced in the areas of memory, attention and executive functioning [47]. Low intelligence may be an independent risk factor for schizophrenia rather than the manifestation of a single underlying pathogenetic process.

## Conclusion

This study confirms the significant role of familial liability in the pathogenesis of VEOS/EOS, and opens more specific questions about the phenotypic patterns of familial transmission. On the other hand the study doesn't confirm the association between obstetric complications pre, peri and postpartum and VEOS/EOS. Moreover the study shows a statistical association between childhood developmental abnormalities and EOS/VEOS, with particular reference to early relational skills and cognitive ability, in support of the neurodevelopmental model of disease.

## Abbreviations

EOS: Early Onset Schizophrenia; VEOS: Very Early Onset Schizophrenia; DSM IV-TR: Diagnostic and Statistical Manual for Mental Disorders, IV Edition-Text Revision; ICHD-II: International Classification of Headache Disorders-II edition; K-SADS-PL: Kiddie-Schedule for Affective Disorders and Schizophrenia-Present and Lifetime Version; EEG: electroencephalograph; MRI: magnetic resonance images; WISC-R: Wechsler Intelligence Scale for Children-Revised; IQ: Intelligence Quotient; OR: Odds Ratio; CI: Confidential Interval; 1H-MRS: proton magnetic resonance spectroscopy; NAA: N-acetylaspartate; Cr: creatine plus phosphocreatine; Cho: choline.

## Competing interests

The authors declare that they have no competing interests.

## Authors' contributions

FM: participated in the design of the study and has been involved in revising critically of the manuscript; MGP: carried out acquisition of data, drafted the manuscript and has been involved in revising it critically; PAL and EL: has contributed in the acquisition of data and helped to draft the manuscript; ADG and OT: has contributed in the acquisition of data; DM: participated in the design of the study and performed the statistical analysis; LM: conceived the study and coordinated the study group. All Authors read and approved the final manuscript

## Authors' information

**FM **Professor Psychiatry, MD

**MGP **Psychiatrist, MD, PhD student

**PAL **Psychologist, PhD student

**OT **Professor Psychiatry, MD

**ADG **Professor Child Neuropsychiatry, MD

**EL **Child Neuropsychiatrist, MD, PhD student

**DM **MD

**LM **Professor Child Neuropsychiatry MD

## Pre-publication history

The pre-publication history for this paper can be accessed here:

http://www.biomedcentral.com/1471-244X/11/60/prepub
